# Anti-Neuroinflammatory Effects of *Arecae pericarpium* on LPS-Stimulated BV2 Cells

**DOI:** 10.3390/cimb46010056

**Published:** 2024-01-19

**Authors:** Hyeon-gyu Cho, Dong-Uk Kim, Jin-Young Oh, Sung-Joo Park, Bitna Kweon, Gi-Sang Bae

**Affiliations:** 1 Department of Pharmacology, School of Korean Medicine, Wonkwang University, Iksan 54538, Republic of Korea; hgyucho@gmail.com (H.-g.C.); ckck202@naver.com (D.-U.K.); vivid3665@naver.com (J.-Y.O.); 2 Hanbang Cardio-Renal Syndrome Research Center, School of Korean Medicine, Wonkwang University, Iksan 54538, Republic of Korea; parksj08@hanmail.net; 3 Department of Herbology, School of Korean Medicine, Wonkwang University, Iksan 54538, Republic of Korea; 4 Research Center of Traditional Korean Medicine, Wonkwang University, Iksan 54538, Republic of Korea

**Keywords:** *Arecae pericarpium*, BV2 cells, anti-neuroinflammation, cytokine

## Abstract

*Arecae pericarpium* (AP), the fruit peel of the betel palm, is a traditional Oriental herbal medicine. AP is used to treat various diseases and conditions, such as ascites, edema, and urinary retention, in traditional Korean medicine. Recent studies have demonstrated its anti-obesity and antibacterial effects; however, its anti-neuroinflammatory effects have not yet been reported. Therefore, we investigated the anti-neuroinflammatory effects of AP on lipopolysaccharide (LPS)-stimulated mouse microglia in this study. To determine the anti-neuroinflammatory effects of AP on BV2 microglial cells, we examined the production of nitric oxide (NO) using Griess assay and assessed the mRNA expression levels of inflammatory mediators, such as inducible NO synthase (iNOS) and cyclooxygenase (COX)-2, and pro-inflammatory cytokines, such as interleukin (IL)-1β, IL-6, and tumor necrosis factor (TNF)-α, using a real-time reverse transcription-polymerase chain reaction. Furthermore, we determined the levels of mitogen-activated protein kinases and IκBα via Western blotting to understand the regulating mechanisms of AP. AP treatment decreased NO production in LPS-stimulated BV2 cells. Additionally, AP suppressed the expression of iNOS and COX-2 and the production of pro-inflammatory cytokines. AP also inhibited the activation of p38 and nuclear factor-kappa B (NF-κB) in LPS-stimulated BV2 cells. Therefore, AP exerts anti-neuroinflammatory effects via inactivation of the p38 and NF-κB pathways.

## 1. Introduction

The socioeconomic costs of neurodegenerative diseases such as Alzheimer’s disease, Parkinson’s disease, and multiple sclerosis are increasing worldwide [1]. However, the pathogenesis of neurodegenerative diseases is still not clearly understood. Recently, neuroinflammation has been considered as a risk factor for various neurodegenerative diseases [2]. Neuroinflammation is an inflammatory response that occurs in the central nervous system, such as the brain or spinal cord, due to pathogens, nerve damage, or trauma [3,4]. This neuroinflammation can have a beneficial effect by removing damaged or dead cell debris and aiding recovery. However, when the balance of the immune response is lost, it has a negative effect on other tissues, causing damage or inhibiting regeneration [5]. If this condition continues, damage and cell death may occur in nerve cells, which may lead to a loss of function and neurodegeneration.

The central nervous system is composed of neuronal and non-neuronal cells. Microglia, a type of non-neuronal cell, act as the resident macrophages in the central nervous system and are involved in the progression of neuroinflammation [6]. Microglial cells aid in the development of nerve cells, contribute to the homeostasis of the central nervous system by removing any defective neurons and synapses, and protect the nerve cells from pathogen infiltration and abnormal protein accumulation [7,8,9]. However, the excessive activation of microglial cells leads to the overexpression of pro-inflammatory cytokines, such as interleukin (IL)-1β, IL-6, and tumor necrosis factor (TNF)-α, and inflammation-inducing factors, such as inducible nitric oxide synthase (iNOS) and cyclooxygenase (COX)-2, which damage the neurons and cause neuroinflammation [10].

*Arecae pericarpium* (AP) is the fruit peel of the betel palm (*Areca catechu* Linné), a member of the *Palmae* family; immature fruits of this family are boiled and peeled. It is mainly native to China and South Korea and Southeast Asia, and its parts are widely used for medicinal purposes [11]. AP is used to treat various diseases and conditions, such as ascites, constipation, edema, and urinary retention, in traditional Korean medicine. As AP and Arecae semen are the fruit peels and seeds of the betel palm (*A. catechu* Linné), respectively, they have similar efficacies; however, the efficacy of AP (fruit peels) is weaker than that of Arecae semen (seeds) [12]. Although previous studies have investigated various effects, such as anti-inflammatory, anti-oxidant, anti-cancer, and anti-aging effects, of seeds, studies on AP have been limited to its anti-obesity, anti-bacterial, and anti-oxidant effects [13,14,15,16,17]. Therefore, in this study, we investigated the anti-neuroinflammatory effects of the AP extract in a lipopolysaccharide (LPS)-induced neuroinflammation model in BV2 mouse microglial cells. We analyzed the effects of AP extract on the production of nitric oxide (NO) and expression levels of iNOS, COX-2, and pro-inflammatory cytokines (IL-1β, IL-6, and TNF-α). Additionally, we evaluated the effects of the AP extract on mitogen-activated protein kinase (MAPK) and nuclear factor kappa B (NF-κB) levels.

## 2. Materials and Methods

### 2.1. Sample Preparation

For this study, AP was purchased from Gwangmyeongdang Pharmaceutical (lot.CK21-G072-1-342, Ulsan, Republic of Korea), which has received a Good Manufacturing Practice certification. AP is an herbal medicine registered in the Korean Pharmacopoeia and can be used in Korea without any regulations [18]. AP extract was prepared by boiling 100 g AP in 1 L distilled water for 150 min. Thereafter, the extract was freeze-dried to obtain a powder (11.2 g), dissolved in distilled water, and filtered. The filtrate was stored at 4 °C until use. We previously reported that the aqueous AP extract contains approximately 0.604% of 4-hydroxybenzoic acid and 0.388% of (+)-catechin as bioactive compounds [19].

### 2.2. Cell Culture

BV2 microglial cells were kindly donated by Professor Dong-sung Lee (Josun University, Kwangju, Republic of Korea). The cells were cultured in the Roswell Park Memorial Institute-1640 medium (Gibco, Thermo Fisher Scientific, Waltham, MA, USA) with 10% fetal bovine serum, 100 U/mL penicillin, 100 g/mL streptomycin and were incubated at 37 °C and 5% CO_2_. Cells were grown to 70–80% confluency and used in experiments.

### 2.3. Cell Viability Assay

BV2 cells were seeded in a 24-well plate at a density of 2 × 10^5^ cells/well. Then, various concentrations of the AP extract (0.05, 0.1, 0.15, 0.2, 0.5, 1, and 2 mg/mL) were added, and cells were incubated for 24 h. Thereafter, a water-soluble tetrazolium (WST) assay was conducted using a kit (Abcam, Cambridge, UK), according to the manufacturer’s instructions. Absorbance at 450 nm was measured using the Spectramax plus 384 (Molecular devices, San Jose, CA, USA).

### 2.4. NO Assay

NO concentration was determined using the Griess reagent (1% sulfanilamide, 0.1% N-1-naphthylenediamine dihydrochloride, and 45% phosphoric acid), which detects NO_2_^−^ present in the culture medium. BV2 cells were seeded in a 24-well plate at a density of 2 × 10^5^ cells/well, pre-treated with the AP extract (0.05, 0.1, and 0.15 mg/mL) or curcumin (20 μM; positive control) for 1 h, and co-treated with LPS (1 μg/mL) for 24 h. Equal volumes of the supernatant and Griess reagent were mixed at room temperature. Finally, the absorbance of the reaction mixture was measured at 540 nm using a spectrophotometer.

### 2.5. Real-Time Reverse Transcription-Polymerase Chain Reaction (RT-PCR)

To extract the total RNA, BV2 cells were seeded in a 6-well plate at a density of 1 × 10^6^ cells/well. The AP extract (0.05, 0.1, and 0.15 mg/mL) or curcumin (20 μM; positive control) was pre-treated for 1 h and co-treated with LPS (1 μg/mL) for 6 h. Then, the supernatant was removed, and the cells were lysed using the Easy-Blue RNA extraction kit (iNTRON Biotechnology, Sungnam, Republic of Korea). RNA purity was confirmed using a Gene Quant Pro RNA Calculator (Biochrom, Inc., Cambridge, UK). RNA was reverse-transcribed to cDNA using the ReverTra Ace qPCR RT Kit (Toyobo, Osaka, Japan). SYBR quantitative RT-PCR was performed using the ABI StepOne Plus detection system (Applied Biosystems, Thermo Fisher Scientific, Inc., Waltham, MA, USA), according to the manufacturer’s instructions. The PCR cycling conditions were as follows: 95 °C for 3 min; and 45 cycles of 95 °C for 10 s, 60 °C for 10 s, and 72 °C for 20 s. For each sample, a triplicate test and a control reaction without reverse transcriptase were analyzed to evaluate the expression of the gene of interest and control variations in the reactions (Table 1).

### 2.6. Western Blotting

BV2 cells were seeded in a 6 cm dish at a density of 1 × 10^6^ cells/dish and the AP extract (0.1 mg/mL) was added to the cells 1 h before incubation with or without LPS (1 μg/mL) for 0, 15, 30, or 60 min. Then, the supernatant was removed, and the cells were lysed with the radioimmunoprecipitation assay buffer on ice (cat. no. IBS-BR004; iNtRON Biotechnology, Sungnam, Republic of Korea). The lysates were boiled in 62.5 mM Tris-HCl buffer (pH 6.8) containing 2% sodium dodecyl sulfate, 20% glycerol, and 10% 2-mercaptoethanol. Then, proteins were separated into a 10% sodium dodecyl sulfate-polyacrylamide gel and transferred onto a nitrocellulose membrane. The membrane was blocked with 5% skim milk in phosphate-buffered saline with Tween 20 for 2 h at room temperature, followed by incubation with primary antibodies (anti-phosphorylated p38 [1:1000; 9211], extracellular signal-regulated kinase (ERK)-1/2 [1:1000; 9101], and c-Jun N-terminal kinase (JNK) [1:1000; 9251], inhibitory κ Bα (Iκ Bα) [1:1000; 9212] antibodies) overnight at 4 °C. After washing thrice, the membrane was incubated with secondary antibodies horseradish peroxidase (HRP)-conjugated goat and anti-rabbit IgG [1:5000; sa002-500]) for 1 h at room temperature. Proteins were visualized using an enhanced chemiluminescence detection system (Amersham, Buckinghamshire, UK), according to the manufacturer’s protocol.

### 2.7. Statistical Analysis

All experiments were conducted more than three times. Based on the average value, the results were expressed as the mean ± standard deviation. All experimental results were analyzed using one-way analysis of variance with the SPSS analysis program version 10.0 (SPSS Inc., Chicago, IL, USA). Statistical significance was set at *p* < 0.05.

## 3. Results

### 3.1. Effect of the AP Extract on BV2 Cell Viability

**Figure 1:**
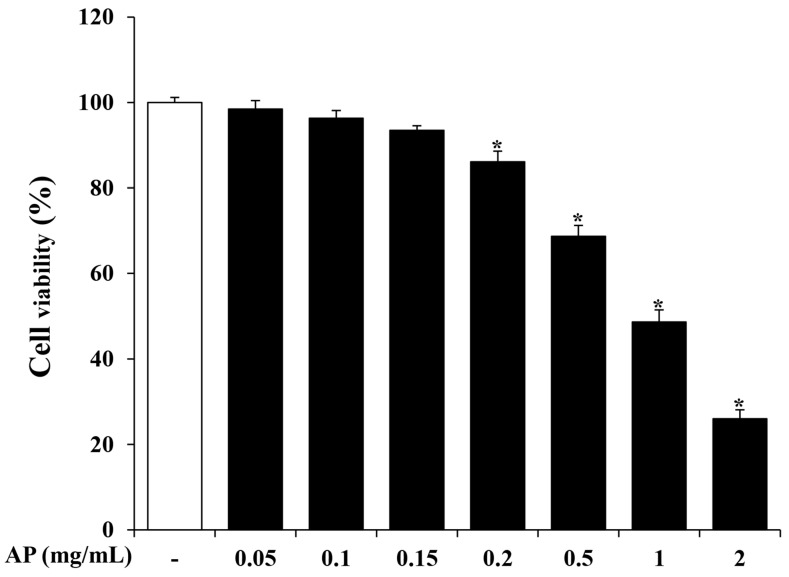
Cytotoxic effects of *Arecae pericarpium* (AP) on BV2 cells. BV2 cells were incubated with various concentrations of AP (0.05, 0.1, 0.15, 0.2, 0.5, 1, and 2 mg/mL). After 24 h, cell viability was measured using water soluble tetrazolium (WST) assay, as described in the Section 2. Results are presented as the mean ± standard error of the mean (S.E.M). Results are representative of three independent experiments. * *p* < 0.05 vs. saline treatment alone.

A WST assay was conducted to determine the appropriate AP extract concentration. Cell viability was measured after treatment with 0.05, 0.1, 0.15, 0.2, 0.5, 1, and 2 mg/mL of the AP extract and incubated for 24 h. As shown in Figure 1, cell viability was not affected by ≤0.15 mg/mL but significantly decreased with ≥0.2 mg/mL of the AP extract. The IC50 concentration of AP extract was calculated as 1.01 mg/mL. Therefore, ≤0.15 mg/mL of the AP extract was selected for subsequent experiments (Figure 1).

### 3.2. Effect of the AP Extract on NO Production in LPS-Stimulated BV2 Cells

**Figure 2:**
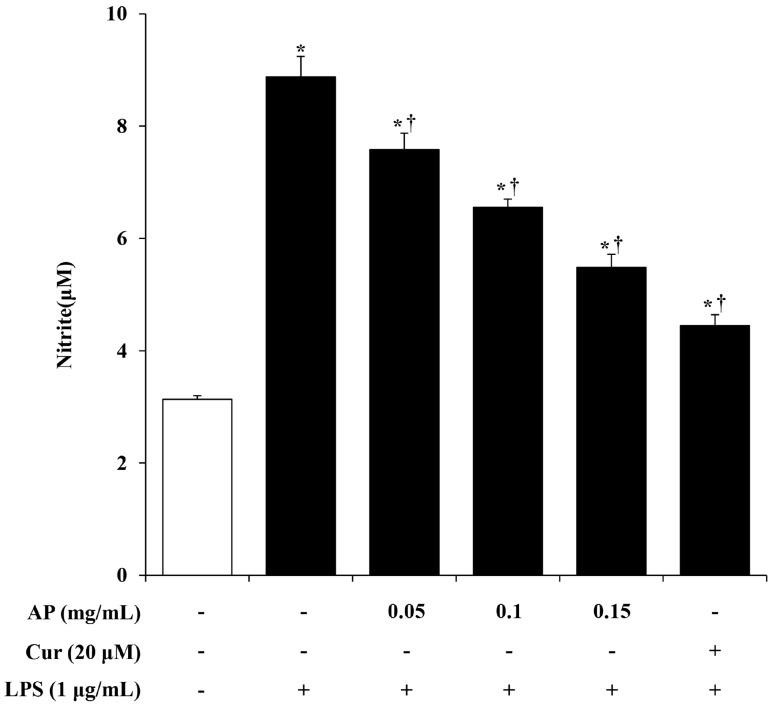
Effect of AP on nitric oxide (NO) production in lipopolysaccharide (LPS)-stimulated BV2 cells. BV2 cells were pre-treated with various concentrations of AP (0.05, 0.1, and 0.15 mg/mL) or curcumin (20 μM; positive control) for 1 h, followed by incubation with LPS (1 μg/mL) for 24 h. Then, NO concentration in the supernatant was determined using the Griess assay, as described in the Section 2. Results are presented as the mean ± S.E.M. Results are representative of three independent experiments. * *p* < 0.05 vs. saline treatment alone; † *p* < 0.05 vs. LPS treatment alone.

Microglia cells are activated by various factors and secrete NO, which promotes the inflammatory response. However, the excessive production and secretion of NO causes damage to nerve cells, leading to neuroinflammation [20]. NO concentration was measured using the Griess assay to determine the anti-inflammatory effects of the AP extract. Based on the results of another study showing the anti-neuroinflammatory effect of curcumin in the LPS-stimulated BV2 cell model, curcumin was used as a positive control in this study [21]. BV2 cells were pre-treated with the AP extract (0.05, 0.1, and 0.15 mg/mL) or curcumin (20 μM) for 1 h and co-treated with LPS (1 μg/mL) for 24 h. NO levels in the cell culture medium were significantly elevated after LPS treatment compared to the control cell group. However, treatment with AP extract reduced NO production in a dose-dependent manner. (Figure 2). The inhibitory effect of AP on NO production was comparable to curcumin (the positive control).

### 3.3. Effects of the AP Extract on iNOS and COX-2 Expression Levels in LPS-Stimulated BV2 Cells

**Figure 3:**
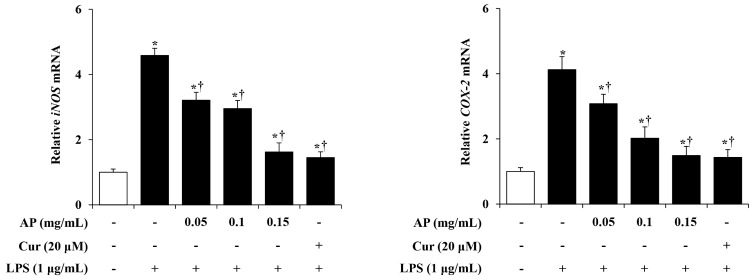
Effects of AP on mRNA expression levels of inducible nitric oxide synthase (*iNOS*) and cyclooxygenase (*COX*)-*2* in LPS-stimulated BV2 cells. BV2 cells were pre-treated with various concentrations of AP (0.05, 0.1, and 0.15 mg/mL) or curcumin (20 μM) for 1 h, followed by incubation with LPS (1 μg/mL) for 6 h. Then, mRNA levels of *iNOS* and *COX-2* were measured using real-time polymerase chain reaction (PCR). Results are presented as the mean ± S.E.M. Results are representative of three independent experiments. * *p* < 0.05 vs. saline treatment alone; † *p* < 0.05 vs. LPS treatment alone.

iNOS and COX-2 are important enzymes that mediate the inflammatory process. Excessive upregulation of iNOS or COX-2 is involved in the pathophysiology of several inflammatory disorders [22]. Therefore, to evaluate the effects of the AP extract on inflammation-inducing factors, such as iNOS and COX-2, we examined the mRNA levels of iNOS and COX-2 using RT-PCR. BV2 cells were pre-treated with the AP extract (0.05, 0.1, and 0.15 mg/mL) or curcumin (20 μM) for 1 h and stimulated with LPS (1 μg/mL) for 6 h. The mRNA levels of iNOS and COX-2 were higher in the LPS-stimulated BV2 cell group than in the control cells. However, treatment with the AP extract inhibited this effect in a dose-dependent manner (Figure 3). The inhibitory effect of AP on iNOS and COX-2 was comparable to curcumin (the positive control).

### 3.4. Effect of the AP Extract on Pro-Inflammatory Cytokine Production in LPS-Stimulated BV2 Cells

**Figure 4:**
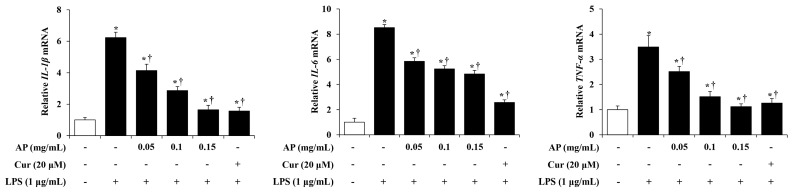
Effects of AP on mRNA expression levels of interleukin (*IL*)*-1β*, *IL-6*, and tumor necrosis factor (*TNF*)-*α* in LPS-stimulated BV2 cells. BV2 cells were pre-treated with various concentrations of AP (0.05, 0.1, and 0.15 mg/mL) or curcumin (20 μM) for 1 h, followed by incubation with LPS (1 μg/mL) for 6 h. Then, mRNA levels of *IL-1β*, *IL-6*, and *TNF-α* were measured using real-time PCR. Results are presented as the mean ± S.E.M. Results are representative of three independent experiments. * *p* < 0.05 vs. saline treatment alone; † *p* < 0.05 vs. LPS treatment alone.

Cytokines are small polypeptides, and most cytokines regulate cell activities such as cell growth, differentiation, and survival at a low concentration [22]. In particular, pro-inflammatory cytokines (IL-1β, IL-6, and TNF-α) produced by activated microglia are generally intended to protect the central nervous system, but excessively produced pro-inflammatory cytokines actually damage central nervous system tissue [22]. Therefore, we examined the mRNA levels of proinflammatory cytokines such as IL-1β, IL-6, and TNF-α using RT-PCR. BV2 cells were pre-treated with the AP extract (0.05, 0.1, and 0.15 mg/mL) or curcumin (20 μM) for 1 h and stimulated with LPS (1 g/mL) for 6 h. The mRNA levels of IL-1β, IL-6, and TNF-α were significantly increased in the LPS-stimulated BV2 cells group compared to the control cells group. However, AP extract reduced the mRNA levels of pro-inflammatory cytokines in a concentration-dependent manner, and, in particular, IL-1β and TNF- α were reduced to levels similar to those of the control cells group (Figure 4). The inhibitory effect of AP on pro-inflammatory cytokines was comparable to curcumin (the positive control).

### 3.5. Effect of AP Extract on MAPK and NF-κB Pathways in LPS-Stimulated BV2 Cells

**Figure 5:**
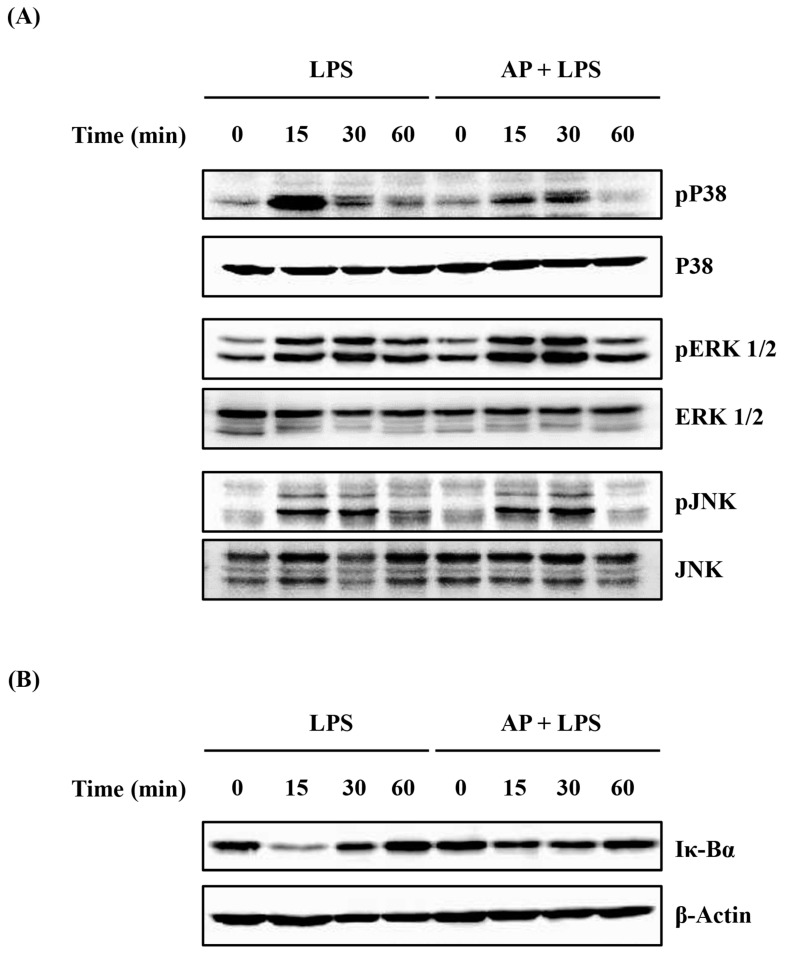
Effects of AP on mitogen-activated protein kinase (MAPK) phosphorylation and Iκ-Bα degradation in LPS-stimulated BV2 cells. BV2 cells were pre-treated with AP (0.15 mg/mL) for 1 h and incubated with LPS (1 μg/mL) for the indicated period. Then, (**A**) MAPK phosphorylation and (**B**) Iκ-Bα degradation were evaluated through Western blotting, as described in the Section 2. Results are representative of three independent experiments.

MAPK and NF-κB pathways played a major role in regulating inflammation in BV2 cells stimulated with LPS [23,24]. To determine the mechanism underlying the anti-inflammatory effects of the AP extract, we evaluated the effect of the AP extract on the activation of MAPK and NF-κB pathways, key pathways involved in the production of pro-inflammatory cytokines, via Western blot analysis. BV2 cells were pre-treated with the AP extract (0.15 mg/mL) for 1 h and stimulated with LPS for 0, 15, 30, and 60 min. As shown in Figure 5, LPS treatment triggered the phosphorylation of MAPKs and degradation of Iκ-Bα in BV2 cells. Interestingly, AP extract inhibited the phosphorylation of p38 and degradation of Iκ-Bα, but not ERK-1/2 and JNK.

## 4. Discussion

Betel nut is considered the most addictive substance after cigarettes, alcohol, and coffee [25]. Up to 10% of the world’s population is known to chew betel nut, especially in Southeast Asia and the Pacific [26]. However, betel nut chewing is closely related to the incidence of oral cancer, and arecoline, an alkaloid contained in betel nut, is believed to be involved in the development of oral cancer [27,28]. Also, the International Agency for Research on Cancer, an affiliate of the World Health Organization, recently designated arecoline as a carcinogen. Therefore, there are concerns regarding the use of AP and Arecae semen as herbal medicines. Typically, the immature pulp, either alone or wrapped in leaves, is used for chewing betel nut, but this is not related to AP. Additionally, other studies indicate that arecoline, a component of betel nut, is sensitive to heat, and its content decreases at higher temperatures [29,30]. When comparing the arecoline content in AP and Arecae semen, which are utilized in herbal medicine, almost no arecoline was detected in AP [31]. AP extract used in this study undergoes a two-step heating process (boiling and drying) during collection. Given that the boiling process is also included in the preparation of the AP extract, it is presumed that the arecoline content has been significantly reduced. Nevertheless, as AP extracts contain numerous other components, conducting single and repeated dose toxicity tests in laboratory animals is considered necessary to assess the toxicity or carcinogenicity of AP extract.

Neurodegenerative diseases are most characterized by a loss of central nervous system neurons, resulting in symptoms such as a loss of physical function and decline in cognition and memory. The number of patients with Alzheimer’s disease and Parkinson’s disease, which are representative neurodegenerative diseases, is increasing every year and these conditions are more common in the elderly [32]. Neurodegenerative diseases are diseases that not only reduce the quality of life of patients but also threaten their lives. Currently, several drugs are used to treat neurodegenerative diseases, but most of them simply help to improve symptoms, and so the development of new treatments or drugs is urgently needed [33].

Microglial cells are important cells in the central nervous system that respond to tissue damage during the early stages. However, the excessive activation of microglial cells results in the secretion of inflammatory mediators, such as pro-inflammatory cytokines, reactive oxygen species, NO, and PGE_2_, which damage tissues and cause neuroinflammation, resulting in neurodegenerative diseases [34]. Therefore, the regulation of inflammatory mediators secreted by hyperactivated microglia is emerging as a new treatment method for neurodegenerative diseases [35]. To date, anti-inflammatory effects of the seeds and leaves of the betel palm (*A. catechu* Linné) have been reported [36,37]; however, the anti-inflammatory and neuroprotective effects of its fruit peel (AP) remain unknown. Since Arecae semen and AP are considered to have similar pharmacological efficacy in Korean medicine [8], we investigated the anti-inflammatory effects of the AP extract and its mechanism of action in LPS-stimulated BV2 cells.

NO is produced by various cells and plays important roles in many physiological and pathological processes, such as stimulating hormone release, dilating blood vessels, and acting as a signaling molecule [38]. In particular, NO plays a key role as a mediator in the inflammatory process. While NO generally has an anti-inflammatory effect under normal conditions, it acts as a pro-inflammatory mediator under certain conditions. NO synthase (NOS), which has three isoforms, endothelial, neuronal, and inducible, converts l-arginine to NO. Among them, iNOS expression is increased by bacterial endotoxins and inflammatory stimuli and is involved in the inflammatory response [39,40]. However, excessive secretion of NO in the central nervous system is related to neurological diseases, causing the damage and inflammation of nerve cells and disrupting the blood–brain barrier [41]. Here, we investigated the effect of the AP extract on NO production in LPS-stimulated BV2 cells. First, cell viability was measured to determine the concentration range of the AP extract in BV2 cells. As shown in Figure 1, there was no cytotoxicity with up to 0.15 mg/mL of AP extract. Next, we evaluated the production of NO using the Griess reagent. BV2 cells stimulated with LPS secreted significantly more NO, but AP extract suppressed NO secretion in a dose-dependent manner (Figure 2). This effect was mediated by a reduction in the expression of mRNA levels of iNOS and COX-2, which are involved in the production of the inflammatory mediators, NO and PGE2, respectively (Figure 3).

Cytokines are membrane-bound protein-based cell signaling molecules that facilitate intercellular communication during immune responses and stimulate cell transport to inflammatory and infected sites [42]. Pro-inflammatory cytokines, such as IL-1 β, IL-6, and TNF-α, are mainly produced by activated macrophages and enhance the inflammatory responses [43]. Inflammatory responses are defensive mechanisms of the body that respond to harmful stimuli, such as pathogens, toxic compounds, and damaged cells; however, the overexpression of pro-inflammatory cytokines causes excessive inflammatory reactions and damages the normal cells [44]. In particular, excessive inflammatory responses in the central nervous system significantly affect the progression of neurodegenerative diseases, such as Alzheimer’s and Parkinson’s diseases [45]. Therefore, the regulation of pro-inflammatory cytokines may be a target for the treatment of neurodegenerative diseases. In this study, we evaluated the mRNA levels of proinflammatory cytokines via RT-PCR. In LPS-stimulated BV2 cells, mRNA levels of major proinflammatory cytokines such as IL-1β, IL-6, and TNF-α were increased. However, AP extract suppressed these increases, and, in particular, IL-1β and TNF-α were decreased to a level almost similar to that of the control group (Figure 4).

MAPK and the NF-κB pathway regulate various cellular functions, such as cell proliferation, division, apoptosis, inflammation, and stress response [46,47]. MAPKs are involved in three major pathways, p38, ERK-1/2, and JNK, which are activated in the cell to reveal their functions. NF-κB is bound to Iκ-Bα in the cytoplasm; when Iκ-Bα is phosphorylated and degraded by various stimulating sources, NF-κB moves to the nucleus and acts as a transcription factor [48,49]. These pathways are easily activated by stimulating factors, such as LPS, cytokines, chemokines, ROS, promoting the production of inflammatory mediators and regulating inflammatory responses [49,50]. Therefore, we evaluated the activation of MAPKs and NF-κB pathways in LPS-stimulated BV2 cells to determine the anti-inflammatory mechanism of the AP extract. LPS induced the phosphorylation of p38, JNK, and ERK-1/2 and the degradation of Iκ-Bα in BV2 cells. But, AP extract inhibited the phosphorylation of p38 and inhibited the degradation of Iκ-Bα in LPS-stimulated BV2 cells (Figure 5). This finding suggests that the AP extract has an anti-neuroinflammatory effect via the inhibition of the p38 MAPK and NF-κB pathways.

We previously reported that the AP extract contains 4-hydroxybenzoic acid and (+)-catechin via chemical profiling using high-performance liquid chromatography [19] (Figure S1). Many studies have reported the beneficial effects of 4-hydroxybenzoic acid (4-HBA) against neuronal and acute inflammation [51,52,53]. As a metabolite of anthocyanin [51], 4-HBA exerts inhibitory effects on neuronal cell death against glutamate-induced excitotoxicity but not neuronal inflammation. Based on a previous study [51], we predicted that the active compound responsible for the anti-neuroinflammatory activity of the aqueous AP extract was not 4-HBA. Many studies have reported that (+)-catechin inhibits neuro-inflammation [54,55,56,57]. Similar to a previous report on (+)-catechin in BV2 cells [57], aqueous AP extract inhibited the increase in inflammatory mediator and cytokine levels via the deactivation of the p38 and NF-κB pathways in this study. Additionally, in the neuroinflammation study, whether the ingredients used in the experiment can pass through the blood–brain barrier (BBB) represents a very important issue. BBB is a semi-permeable barrier that exists in the microvessels of the central nervous system and protects the central nervous system from toxins and pathogens in the blood [58]. However, most small-molecule drugs and therapeutics are also blocked from accessing the brain by the BBB [59]. Because of this, various attempts are being made to allow drugs to cross the BBB and work on the central nervous system. According to a previous study, (+)-catechin passed through the BBB in two different BBB cell line models [60]. Summarizing these results, it is believed that (+)-catechin contained in AP extract can cross the BBB and reach the central nervous system and exert anti-neuroinflammatory effects; however, additional studies would be required to substantiate this possibility.

In this study, we explored the anti-neuroinflammatory effects of AP extract; however, there are several limitations. The investigation was solely conducted using BV2 cells, a mouse microglial cell line. The absence of experiments performed on human cell lines or in vivo studies involving more intricate and diverse biological processes could be a limitation of this study. Hence, further experiments addressing these aspects are deemed necessary.

## 5. Conclusions

Though this study, we demonstrated, for the first time, that AP extract has anti-neuroinflammatory effects in LPS-stimulated BV2. AP extract inhibited the production of inflammatory mediators such as NO, inflammatory regulating enzymes such as iNOS and COX-2, and pro-inflammatory cytokines such as IL-1 β, IL-6, and TNF-α in LPS-stimulated BV2. And these results were found because the AP extract inhibited p38, which is one of the MAPK and NF-κB signaling pathways. Taken together, AP may be used as a preventive and therapeutic agent for neurodegenerative diseases caused by neuroinflammation.

## Figures and Tables

**Table 1:** Sequences of primers used for real-time polymerase chain reaction (PCR).

Gene	Primer
*iNOS* (F)	5′-GTT GAA GAC TGA GAC TCT GG-3′
*iNOS* (R)	5′-GAC TAG GCT ACT CCG TGG A-3′
*COX-2* (F)	5′-GGT GGC TGT TTT GGT AGG CTG-3′
*COX-2* (R)	5′-GGG TTG CTG GGG GAA GAA ATG-3′
*IL-1β* (F)	5′-CCT CGT GCT GTC GGA CCC AT-3′
*IL-1β* (R)	5′-CAG GCT TGT GCT CTG CTT GTG A-3′
*IL-6* (F)	5′-CCG GAG AGG AGA CTT CAC AG-3′
*IL-6* (R)	5′-CAG AAT TGC CAT TGC ACA AC-3′
*TNF-α* (F)	5′-AAC TAG TGG TGC CAG CCG AT-3′
*TNF-α* (R)	5′-CTT CAC AGA GCA ATG ACT CC-3′
*GAPDH* (F)	5′-TGT GTC CGT CGT GGA TCT GA-3′
*GAPDH* (R)	5′-TTG CTG TTG AAG TCG CAG GAG-3′

## Data Availability

The data used to support the funding of this study are available from the corresponding author upon request.

## Supplementary Materials

The following supporting information can be downloaded at: https://www.mdpi.com/article/10.3390/cimb46010056/s1, Figure S1. HPLC Analysis of AP extracts.
