# Supplementary figures and images for: Anti-Neuroinflammatory Effects of Arecae pericarpium on LPS-Stimulated BV2 Cells
Source: Curr Issues Mol Biol. 2024 Jan 19;46(1):884–95. doi: 10.3390/cimb46010056 (PMC10814974; doi:10.3390/cimb46010056)

A

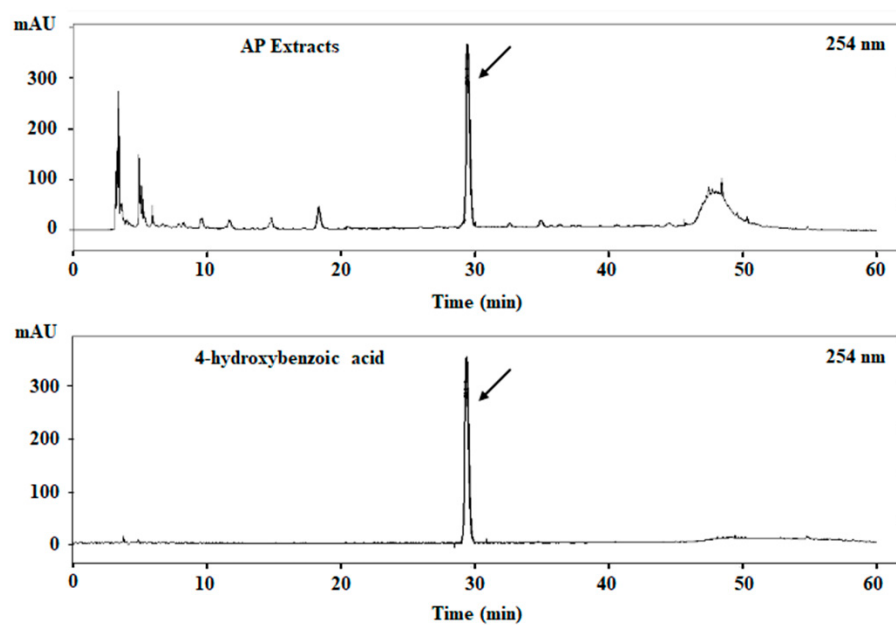

B

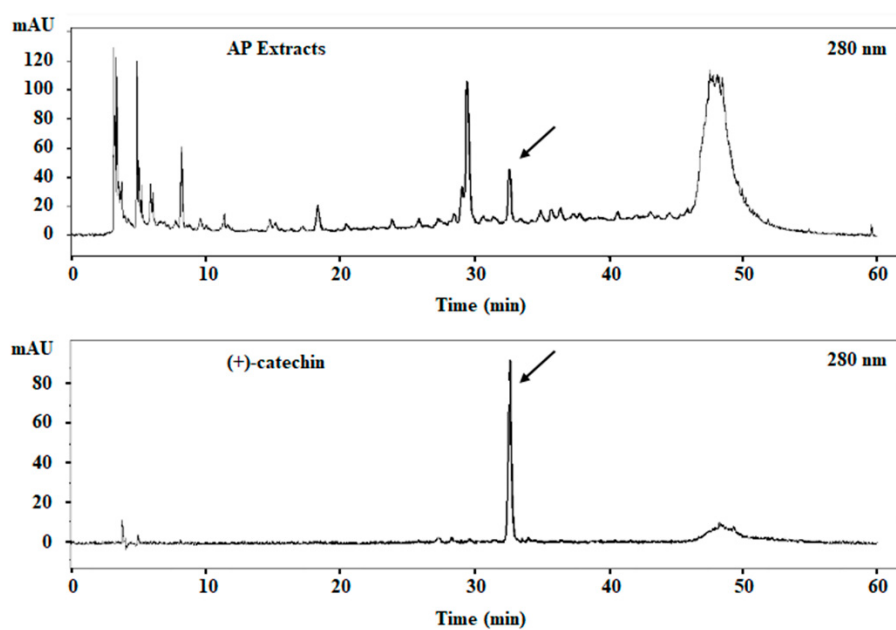

**Figure S1.** HPLC analysis of AP extracts.

Supplement: Supplementary file 1 [file cimb-46-00056-s001.zip › cimb-2812774-supplementary.pdf]
